# CurvAGN: Curvature-based Adaptive Graph Neural Networks for Predicting Protein-Ligand Binding Affinity

**DOI:** 10.1186/s12859-023-05503-w

**Published:** 2023-10-05

**Authors:** Jianqiu Wu, Hongyang Chen, Minhao Cheng, Haoyi Xiong

**Affiliations:** 1 Research Center for Graph Computing, Zhejiang Lab, Yuhang, Hangzhou, 311121 Zhejiang China; 2 Department of Computer Science and Engineering, Hong Kong University of Science and Technology, Jiulongwan, Hongkong, 999077 China; 3 Big Data Lab, Baidu Inc., Haidian, Beijing, 100080 China

**Keywords:** Multiscale curvature, Adaptive graph attention mechanism, Heterophily, Protein-ligand bingding affinity

## Abstract

Accurately predicting the binding affinity between proteins and ligands is crucial for drug discovery. Recent advances in graph neural networks (GNNs) have made significant progress in learning representations of protein-ligand complexes to estimate binding affinities. To improve the performance of GNNs, there frequently needs to look into protein-ligand complexes from geometric perspectives. While the “off-the-shelf” GNNs could incorporate some basic geometric structures of molecules, such as distances and angles, through modeling the complexes as homophilic graphs, these solutions seldom take into account the higher-level geometric attributes like curvatures and homology, and also heterophilic interactions.To address these limitations, we introduce the Curvature-based Adaptive Graph Neural Network (CurvAGN). This GNN comprises two components: a curvature block and an adaptive attention guided neural block (AGN). The curvature block encodes multiscale curvature informaton, then the AGN, based on an adaptive graph attention mechanism, incorporates geometry structure including angle, distance, and multiscale curvature, long-range molecular interactions, and heterophily of the graph into the protein-ligand complex representation. We demonstrate the superiority of our proposed model through experiments conducted on the PDBbind-V2016 core dataset.

**Supplementary Information:**

The online version contains supplementary material available at 10.1186/s12859-023-05503-w.

## Introduction

Protein-ligand binding affinity prediction is a critical step in drug discovery [1]. It allows researchers to identify potential drug candidates and optimize their properties before conducting expensive and time-consuming experiments. The increasing availability of three-dimensional (3D) structural protein data provides a new paradigm for structure-based drug discovery and 3D structural information has been proven to facilitate drug design [2]. Various computational methods have been developed to learn 3D structure information from a protein-ligand complex. These methods range from molecular docking [3–6] to more sophisticated machine learning [1, 7, 8] and deep learning approaches [9].

Docking methods have been widely adopted with a scoring function for binding affinity prediction, but their accuracy also limits the potential applications of docking methods [3, 4]. Traditional machine learning algorithms [7, 8] together with handcrafted features could sometimes deliver decent performance, but they are difficult to scale up due to cost of extensive feature engineering. To model 3D spatial structure, many deep learning approaches [10–12] divide the complex into 3D grid data and apply 3D convolutional neural works(3D CNNs) to extract useful features. These approaches have demonstrated better performance in predicting binding affinity than traditional machine learning-based models. However, the sparsity distribution of atoms in the complex can result in inefficient computations when using a 3D rectangular grid representation [13].

Modeling a protein-ligand complex as a graph where nodes correspond to atoms is a natural and effective approach [14, 15]. Graph neural networks (GNNs) have demonstrated remarkable capabilities in expressing graph structures, and researchers have made considerable efforts to incorporate spatial information to enhance its expression ability. Spatial Graph Convolutional Networks [16, 17] utilize 3D coordinates to model the structure of complexes. However, the output of coordinate-based models can be negatively impacted by rotations of the coordinates. This limitation is addressed by distance-aware GNNs [13, 18], which only take distance into account. But these models may not suffice to accurately model 3D structures for binding affinity predictions. Directional message passing-based GNNs [2, 19] have been proposed to address this limitation. These models incorporate angle and distance information, which has been shown to be crucial in empirical potentials for molecules [20]. While these models offer improved prediction performance, their accuracy have a great potential to be further improved. Since the protein-ligand binding affinity is determined by its absolute binding free energy [21], which is primarily specified by curvature [22], incorporating curvature information into the graph representation is necessary to enhance prediction accuracy.The concept of curvature is closely related to the geometry of a manifold, and some efforts have been made to generalize curvatures for a graph [23, 24]. Based on this generalization, two different curvature-based graph neural networks [25, 26] have been proposed, and they perform well on baseline datasets. Biomolecules often exhibit hierarchical and multiscale structures, which require a multiscale representation to accurately characterize their interactions [27]. It implies multiscale curvature for graph is more suitable. However, incorporating multiscale curvature into GNNs for predicting binding affinity remains an open research question.

Moreover, many studies have recognized the heterogeneity of protein-ligand complex graphs and endeavored to incorporate this heterogeneity into their graph neural networks [2, 28, 29]. Nevertheless, it is often disregarded that the graph is not strictly homophilic, as neighboring nodes may not be similar. Graph neural networks based on the homophily assumption cannot effectively learn heterophily, which is the property where linked nodes have dissimilar features [30, 31]. Therefore, previous studies on binding affinity have failed to capture heterophily.

To address above challenges, we propose a novel Curvature-based Adaptive Graph Neural Network (CurvAGN) for predicting protein-ligand binding affinity. The CurvAGN comprises a curvature block and an adaptive attention guided neural block (AGN). The curvature block assigns edge attributes to include multiscale curvature, and AGN is inspired by SIGN [2] and consists of two parts. The first part, called the polar-inspired adaptive graph attention block (PAGA), uses an adaptive graph attention mechanism [32] to model the 3D spatial structure of the protein-ligand complex by incorporating distance, angle, and curvature information. The adaptive attention mechanism addresses the heterophily in the protein-ligand complex graph. The second part is the pooling block, which is described in [2] and includes the pairwise interactive pooling (PiPool) for leveraging long-range interactions and the output pooling layer for predicting the protein-ligand binding affinity.

Our work makes three main contributions:

- We propose the curvature block that utilizes multiscale curvature to encode edge attributes of biomolecule graphs, effectively capturing the multiscale structure of these biomolecules.
- We find the distance-based complex interaction graph is a heterophilic graph, and further propose the adaptive attention guided neural model (AGN) to capture the heterophily and geometric structure of angles and distances, and and long-range molecular interactions.
- We combine the curvature-based graph neural network and AGN to propose the Curvature-based Adaptive Graph Neural Network (CurvAGN).
- We apply CurcAGN to predicting the protein-ligand binding affinity. We train and validate our model on the publicly available standard PDBbind-v2016 dataset, and show that it outperforms SIGN [2] by 7.5% in RMSE and 9.4% in MAE.

## Related work

### 3D structure GNNs for binding affinity prediction

3D structural GNNs have been used to integrate the 3D structure of protein-ligand complexes into high-level representations, thereby improving the accuracy of binding affinity prediction. Atom coordinate-based GNNs [17] use atomic coordinates directly as node attributes, but they often fail to recognize the same protein-ligand complex due to coordinate variations in different coordinate systems. Distance-based GNNs [13, 33–35] overcome this deficiency by utilizing atomic distances. Angle and distance-based GNNs [2, 19] can enrich geometric information and enhance complex modeling capabilities.

### Ricci curvature for graphs

Ricci curvature is a geometric object that measures the curvature of a Riemannian manifold [36, 37]. Intuitively, if the Ricci curvature is positive, the manifold curves more like a sphere, while negative Ricci curvature results in a more saddle-like curve. In recent years, there has been growing interest in the study of graph curvature, which is a discrete analogue of Ricci curvature. There are two main types of graph curvature: Ollivier Ricci curvature (ORC) and Forman Ricci curvature (FRC). ORC is based on optimal transport theory and captures the geometric properties of a graph [23, 38–43], while FRC is based on the graph Laplacian and captures the algebraic topological properties of a graph [24, 44]. In general, ORC is a more recent and sophisticated measure of curvature than FRC. However, FRC is more widely used because it is easier to compute.

### Persistent graph-curvature-descriptors

Xia et al. propose a persistent graph curvature descriptor to characterize molecular features based on the observation that biomolecules have a hierarchical and multiscale structure [27, 43]. They first filter the edges of the graph by length to remove short edges that are less relevant to the hierarchical structure, and then construct a sequence of subgraphs, where each subgraph is a subset of the next one. They then define a permutation-invariant descriptor function for each subgraph that is related to curvature. This function is designed to be invariant to the order in which the nodes are arranged, so that it can be used to characterize the molecular features of the graph regardless of how the graph is represented. Finally, they arrange the descriptors of each subgraph in sequence, to form the persistent graph curvature descriptor.

### Heterophily-based GNNs

Heterophilic graphs refer to graphs where linked nodes exhibit heterophily, meaning that they have dissimilar features and different class labels [29]. Many real-world graphs, such as transaction networks [45], exhibit heterophily. Recent studies have shown that GNNs do not perform well on heterophilic graphs [46–49]. This is because GNNs are typically designed to learn from homophilic graphs, where linked nodes have similar features and class labels. To address this issue, several GNN designs have been proposed that are specifically tailored for heterophilic graphs. These designs include MixHop [50], MM-DAN [51], BeyondGNN [32], AdaGNN [52], Beyond-GCN [53], and Geom-GCN [54].

Persistent curvature descriptors have been shown to be effective in representing protein-ligand complexes, but they rely on prior knowledge. To overcome this limitation, we developed a multiscale curvature graph neural network that incorporates the multiscale curvature of edges as edge attributes. In addition to the curvature information, the interactions between molecules play a critical role in binding affinity. When modeling a protein-ligand complex as a graph, protein atoms and ligand atoms are connected based on distance, but short distance does not necessarily mean similar features. This leads to the graph not having strict homophily. To capture this important feature, it is natural to utilize heterophily-based models. However, to the best of our knowledge, no heterophily-based GNNs have been used for modeling this complex yet. Therefore, we propose incorporating the adaptive graph attention mechanism [32] into our network.

## Preliminaries

In this section, we introduce some key definitions that will be used in our model and formulas..

### Definition 1

(Complex Interaction Graph [2, 35]) For an protein-ligand complex, let ${\mathcal {V}}^{\text {L}}:=\{a_1^{L},a_2^{L}, \ldots , a_n^{L} \}$ be the ligand atom set, ${\mathcal {V}}^{\text {P}}:=\{a_1^{P},a_2^{P}, \ldots , a_m^{P} \}$ be the protein atom set. We define the complex interaction graph as a direction graph ${\mathcal {G}}_I:=({\mathcal {V}},{\mathcal {E}})$, where the node set is

$$\begin{aligned} {\mathcal {V}}:={\mathcal {V}}^L \cup \{ a_i \in {\mathcal {V}}^{\text {P}}:\exists \, a_j^{L} \in {\mathcal {V}}^{\text {L}}, \Vert c(a_i)- c(a_j^L)\Vert \le d \}, \end{aligned}$$

and the edge set is

$$\begin{aligned} {\mathcal {E}}:=\{(a_i,a_j) \in {\mathcal {V}}\times {\mathcal {V}}:\Vert c(a_i)-c(a_j)\Vert \le d\}. \end{aligned}$$

Here $c(\cdot )$ sents each atom to is 3D coordinate, $\Vert \cdot \Vert$ is an Euclidean distance, and d is a cutoff distance.

### Definition 2

(Edge-oriented Neighbors [2]) In the complex interaction graph ${\mathcal {G}}_I$, for an atom node $a_i$ or a directed edge $e_{ij}$ (i.e., $a_i \rightarrow a_j$), the edge-oriented neighbors $\text {N}_e$ of $a_i$ or $e_{ij}$ are defined as the sets of directed edges $\{e_{ki}, \ldots , e_{li}\}$ which point to the target atom $a_i$ or the target edge $e_{ij}$.

### Definition 3

(Ollivier Ricci Curvature [42]) For a graph ${\mathcal {G}}:=(\text {V}, \text {E})$, given a $\alpha \in [0,1]$, $\alpha$-Ricci-curvature $k_{\alpha }$ of nodes $a_i$ and $a_j$ is defined to be

$$\begin{aligned} k_{\alpha }(a_i,a_j):= 1 - \frac{\text {W}(m_{a_i}^{\alpha },m_{a_j}^{\alpha })}{d(a_i,a_j)}, \end{aligned}$$

where $d(a_i,a_j)$ is the graph distance between two vertices $a_i$ and $a_j$, $m_a^{\alpha }$ is a probability measure defined as

$$\begin{aligned} m_a^{\alpha }(a_k):= {\left\{ \begin{array}{ll} \alpha &{} a_k=a\\ \frac{1-\alpha }{\text {deg}(a)}&{} a_k \in \text {N}(a)\\ 0 &{} otherwise, \end{array}\right. } \end{aligned}$$

and $\text {W}(\cdot ,\cdot )$ is the transportation distance between two probability distributions $m_1$ and $m_2$, is defined by

$$\begin{aligned} \text {W}(m_1,m_2):= \text {inf}_{\text {A}} \sum _{a_1,a_2 \in V}\text {A}(a_1,a_2)d(a_1,a_2). \end{aligned}$$

Here $\text {deg}(\cdot )$ sents each node to its degree, $\text {N}(a)$ is the neighbors of node *a*, and the map $\text {A}: \text {V} \times \text {V} \rightarrow [0,1]$ is a coupling between $m_1$ and $m_2$ such that

$$\begin{aligned} \sum _{a_2 \in \text {V}}\text {A}(a_1,a_2) = m_1(a_1) \qquad \text {and} \qquad \sum _{a_1 \in \text {V}}\text {A}(a_1,a_2) = m_2(a_2). \end{aligned}$$

### Definition 4

(Foramn Ricci Curvature [24]) When a graph ${\mathcal {G}}:=(\text {V}, \text {E})$ is composed of nodes, edges and triangles, otherwise, Forman-Ricci-curvature *F* of an edge $(a_1,a_2) \in \text {E}$ is defined to be

$$\begin{aligned} F(a_1,a_2):= 4 - \text {deg}(a_1) -\text {deg}(a_2) + 3\Delta _{a_1a_2}, \end{aligned}$$

otherwise, it defined to be

$$\begin{aligned} F(a_1,a_2):= 4 - \text {deg}(a_1) -\text {deg}(a_2), \end{aligned}$$

where $\Delta _{a_1a_2}$ is the number of triangular containing the edge $(a_1,a_2)$.

## Curvature-based adaptive graph neural networks

In this section, we present our model, called CurvAGN (Curvature-based Adaptive Graph Neural Network). We begin by giving an overview of the framework, followed by a detail description of each component.

### Overview

**Fig. 1:**
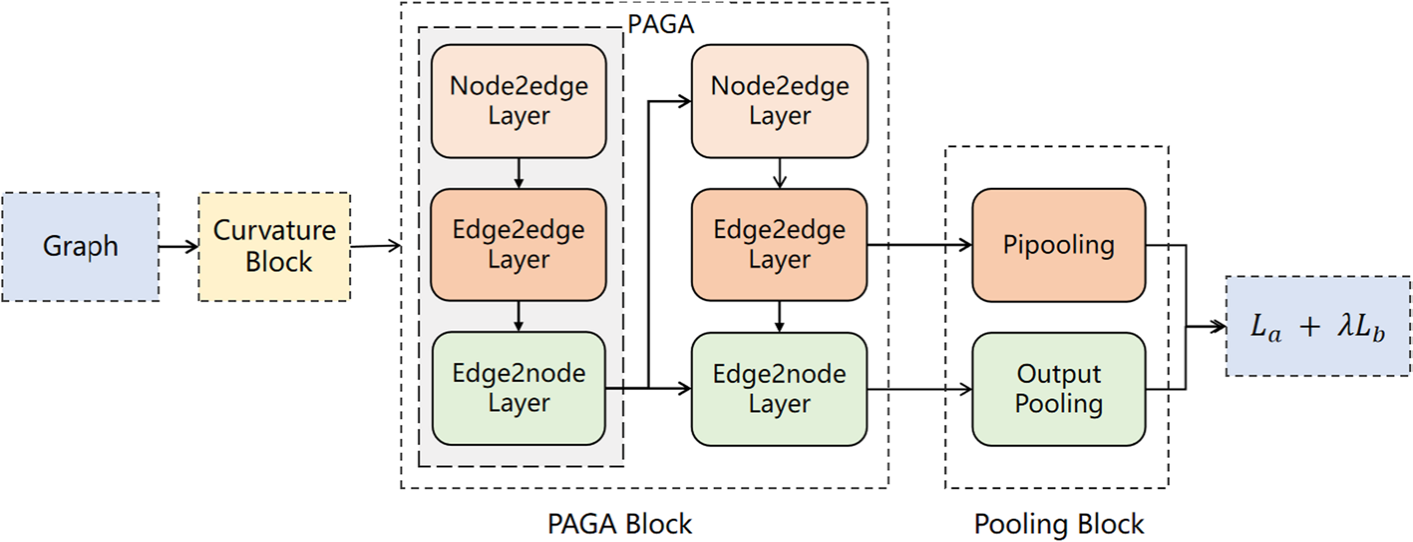
Illustration of the proposed CurvAGN framework. CurvAGN is composed of a curvature block, a PAGA block, and a pooling block. The curvature block encodes multiscale curvature structure and PAGA block incorporates the geometric information including distance, angle, and multiscale curvature, and the heterophily of protein-ligand complex graph into the representation of the complex. The pooling block outputs the co-occurrent frequency of atom pairs by the Pipooling layer and the prediction of the binding affinity by the output pooling layer

The overall framework of CurvAGN is shown in Fig. 1. It takes a complex interaction graph ${\mathcal {G}}_I$ as input and is made up of three blocks: a curvature block, a PAGA block, and a pooling block. The first two blocks, namely the curvature and PAGA block, use a 3D model to capture the geometric structure of the protein-ligand complex interaction graph. Specifically, the curvature block captures the multiscale curvature information of the graph, while PAGA learns the spatial distance and angle information. The pooling block then gets the prediction of the binding affinity and the co-occurrent frequency of atom pairs, such as the Carbon-Carbon co-occurrence frequency.

The PAGA block is composed of multiple PAGA layers, where each layer has a node2edge layer, an edge2edge layer, and an edge2node layer. The node2edge layer utilizes the graph attention mechanism (GAT) to fuse the attribute information of the nodes at both ends of an edge into the edge attributes. The edge2edge layer uses the adaptive GAT to convert the angle information and edge attributes obtained from the first layer into edge representations. Lastly, the edge2node layer employs the adaptive graph attention mechanism to learn the node representations.

The pooling block consists of an output pooling layer and a Pipooling layer. The former generates the binding affinity prediction, while the latter produces the co-occurrent frequency of atom pairs.

### The curvature block

Ricci curvature measures the extent to which a smooth object deviates from being flat. Two different discrete forms of Ricci curvature, Ollivier and Forman, have been incorporated into graph neural networks [25, 26]. Biomolecules often have hierarchical and multiscale structures, requiring a multiscale Ricci curvature to accurately characterize these structures and interactions. Such curvature has been proposed in [27, 43]. However, their curvature descriptor for protein-ligand complexes relies on prior knowledge such as the average and variance of all curvatures, and therefore, is not universally applicable. In contrast, we propose a multiscale curvature for each edge of the graph, making it a more versatile and flexible solution.

Let $\text {dc}: {\mathcal {E}} \rightarrow {\mathbb {R}}$ be a discrete curvature function defined on the edge set ${\mathcal {E}}$ of a complex interaction graph, where ${\mathbb {R}}$ denotes the set of real numbers. The curvature of an edge $e_{ij}$ of the graph is denoted by $\text {dc}(e_{ij})$ or simply $\text {dc}_{ij}$.

To define the multiscale curvature, we first select a sequence of filtration values $\{l_i: l_0< l_1< \cdots < l_{n-1} \}$, then for each $l_k$, construct a subgraph ${\mathcal {G}}^{(k)}$ by removing edges with weight greater than $l_k$ from the original graph and compute the curvature $\text {dc}_{ij}^{(k)}$ of each edge $e_{ij}$ in the subgraph.

The multiscale curvature $\text {fc}_{ij}$ for an edge $e_{ij}$ in the original graph is defined by concatenating the curvatures of the edge in subgraphs according to the order of the sequences, as follows:

$$\begin{aligned} \text {fc}_{ij}:= \Vert _{0 \le k < n} \text {dc}_{ij}^{(k)}, \end{aligned}$$

where $\Vert$ represents concatenation. If the edge $e_{ij}$ is not in the subgraph ${\mathcal {G}}^{(k)}$, we set its curvature $\text {dc}_{ij}$ as zero.

We then apply a dense layer to obtain a multiscale curvature embedding:

1 $$\begin{aligned} f_{ij}:= \text {Softmax}\left( \text {LeakyRelu} (\text {W}_f \cdot \text {fc}_{ij})\right) , \end{aligned}$$

where $\text {W}_f$ is a transformation matrix.

As described in [2], we set a one-hot vector $x_{ij}$ for the weight of edge $e_{ij}$ by taking its integer part. Then the distance embedding for the edge is giving by

2 $$\begin{aligned} d_{ij}:= \text {W}_{d}x_{ij}, \end{aligned}$$

where $\text {W}_{d} \in {\mathbb {R}}^{n_w \times m}$ is a transformation matrix, and $n_w$ represents the dimension of the embedding.

Finally, we define the curvature block as follows:

3 $$\begin{aligned} \text {crt}_{ij}:=\text {ReLU}\left( W_{fd} \cdot [d_{ij}|f_{ij}]\right) , \end{aligned}$$

where $\text {W}_{fd}$ is a transformation matrix and $d_{ij}$ is the distance embedding in Eq. 2.

### The polar-inspired adaptive graph attention block

PAGA is an adaptive graph attention network that models the 3D structure of the complex interaction graph. Compared to PGAL [2], which uses a polar-inspired graph attention block, PAGA focuses on the adaptive graph attention mechanism and the varying dependency of different attributes of a node on a neighboring node. PAGA decomposes the layer into node2edge, edge2edge, and edge2node layers, which allows for a more granular understanding of the structural information.

### The node2edge layer

The node2edge layer passes node information to its edges in order to get the edge representation. In the case of PAGA, we need to add angle information to the 3D model, which requires the transportation of node information to edges. This is done by defining the *l*-th layer of the node2edge layer as follows:

4 $$\begin{aligned} h_{e_{ij}}^{(l)}:=\text {ReLU}\left( \text {W}_{ab}^{(l)} [h_{a_i}^{(l-1)}\Vert h_{a_j}^{(l-1)}\Vert \text {crt}_{ij}]\right) , \end{aligned}$$

here, $\text {W}_{ab}^{(l)}$ is a transformation matrix and $h_{a_i}^{l-1}$ is the $(l-1)$-layer node representation of the node $a_i$.

### The edge2edge layer

The edge2edge layer uses the adaptive graph attention mechanism to update the edge information based on the angles. To apply angle information, we construct a directed line graph and get subgraphs of the line graph by classifying the angles between edges in the original graph.

The directed line graph of the complex interaction graph is a dual graph where the nodes, node attributes, and edge-oriented neighbors of the nodes correspond respectively to the edges, the edge representations, and edge-oriented neighbors of the edges in the original graph. The weight of a directed edge between nodes in the dual is defined as the angle between the corresponding to edges in the complex interaction graph.

To get the subgraph of the line graph, we set *N* angle domains, denoted as $(\frac{180^{\circ }*(q-1)}{N}, \frac{180^{\circ }*q}{N}]$, for $q = 1,2,\ldots , N$. The *q*-th subgraph is the subgraph of the line graph that retains all nodes but only edges of weights in the *q*-th angle domain. We denote the neighbors of a node $e_{ij}$ in the *q*-th subgraph by $\text {N}_e^{q}(e_{ij})$. The aggregation process for the *q*-th local node representation is defined as follows:

5 $$\begin{aligned} m_{ij,q}^{(l)}:= & {} \sum _{e_{ki} \in \text {N}_e^q(e_{ij})} \alpha _{ki,q}^{(l)} \odot h_{e_{ki}}^{(l)} + h_{e_{ij}}^{(l)}, \end{aligned}$$

6 $$\begin{aligned} \alpha _{ki,q}^{(l)}:= & {} \text {tanh}( \text {W}_{e,q}^{(l)}\cdot [h_{e_{ij}}^{(l)}\Vert h_{e_{ki}}^{(l)}] + b_{e,q}^{(l)} ), \end{aligned}$$

where the operator $\odot$ is the Hadamard product, $\text {W}_{e,q}^{(l)}$ is a learnable transformation matrix, and $b_{e,q}^{(l)}$ is a learnable vector. Equation 6 applies the adaptive graph attention mechanism to get an attention vector which is viewed as the concatenation of coefficients of attributes between nodes. And $m_{ij,q}^{(l)}$ in Eq. 5 is the *q*-th local node representation at the *l*-th layer. To obtain the complete node representation, all the local aggregated node representations are combined:

$$\begin{aligned} h_{e_{i,j}}^{(l)}:=[m^{(l)}_{ij,1} \Vert m^{(l)}_{ij,2} \Vert \cdots \Vert m^{(l)}_{ij,\text {N}}]. \end{aligned}$$

For the dual, representation $h^{(i)}_{e_{ij}}$ is also the edge representation in the complex interaction graph.

### The edge2node layer

The node2edge layer incorporates angle information into the edge representation. To further inject the distance and multiscale curvature information into the node representation, we design the edge2node layer based on the adaptive attention mechanism. This is in contrast to the GAT-based distance-aware attention mechanism in [2], which cannot capture heterophily.

Since the feature spaces of edges and nodes are different, we first use learnable parameter matrices $\text {W}_e^{(l)}$ and $\text {W}_a^{(l)}$ convert the representations of nodes and edges to the same space as follows:

7 $$\begin{aligned} {\tilde{h}}_{e_{ij}}^{(l)}:= & {} \text {W}_e^{(l)}\cdot h_{e_{ij}}^{(l)}, \end{aligned}$$

8 $$\begin{aligned} {\tilde{h}}_{a_j}^{(l)}:= & {} \text {W}_a^{(l)}\cdot h_{a_{j}}^{(l-1)}, \end{aligned}$$

Then we define the attention of $e_{ij}$ with respect to $a_j$ as

9 $$\begin{aligned} \beta _{ij}^{(l)}:=\text {tanh}(v_{l}^T\cdot [{\tilde{h}}_{e_{ij}}^{(l)}\Vert {\tilde{h}}_{a_j}^{(l)}\Vert \text {W}_{dr}^{(l)}\text {crt}_{ij}]), \end{aligned}$$

where $v_l^T$ is a parameter vector at the *l*-th layer, and $\text {W}_{dr}^{(l)}$ is the learnable parameter matrix. Finally, we get the multi-head attention version of our edge2node layer by aggregating over all edges $e_{ij} \in \text {N}_e(a_j)$ as follows:

10 $$\begin{aligned} h_{a_{j}}^{(l)}:=\frac{1}{C}\sum _{c=1}^{C}\sum _{e_{ij} \in \text {N}_e(a_j)} \beta _{ij,c}^{(l)} \cdot {\tilde{h}}_{e_{ij},c}^{(l)} + {\tilde{h}}_{a_j,c}^{(l)}, \end{aligned}$$

where *C* is the number of attention heads and $\text {N}_e(a_j)$ is the edge-oriented neighbors of node $a_j$.

Assuming PAGA has *L* polar-inspired adaptive graph attention layers, it yields the node representation $a_j^{(L)}$ for atom $a_j$ and the edge representation $e_{ij}^{(L)}$ between atoms $a_i$ and $a_j$.

### The pooling block

As illustrated in [2], the pooling block is composed of a PiPooling layer and an output pooling layer. The PiPooling layer is designed to capture the long-range intermolecular interactions between the protein and ligand and output poling layer to predict the affinity.

### The PiPooling layer

The PiPooling layer first divides the edges into $|\text {S}_{P}| \times |\text {S}_{L}|$ components, where $\text {S}_{P}$ and $\text {S}_{L}$ be atomic type(number) sets of the protein and its ligand, respectively. For the $(T_k,T_l)$-component, the pooling of edge representations is defined as

11 $$\begin{aligned} h_{kl}:= \sum _{e_{ij}\in {\mathcal {E}}_I}\delta \left( \tau (a_i),T_k\right) \delta \left( \tau (a_j),T_l\right) \text {W}_he_{ij}^{(L)}, \end{aligned}$$

where $W_h$ is a shared parameter, $T_k \in \text {S}_{P},T_l \in \text {S}_{L}$, the map $\tau$ sents each node to its atomic number, $\delta$ is a Kronecker delta function, and ${\mathcal {E}}_I$ is the set containing all the intermolecular edges in the complex ${\mathcal {G}}_I$. The output of PiPool is given by

12 $$\begin{aligned} {\tilde{Z}}_{kl}:=\frac{\text {exp}(q^Th_{kl})}{\sum _{ij} \text {exp}(q^Th_{ij})}, \end{aligned}$$

where $q^T$ is a learnable parameter. And ${\tilde{Z}}_{kl}$ can be considerd an approximation of interaction matrix

13 $$\begin{aligned} Z_{kl}:=\frac{n(T_k,T_l)}{\sum _{(a_i,a_j) \in \text {S}_P \times \text {S}_L} \Theta (d_{\rho } - d_{ij})}, \end{aligned}$$

where $n(T_k,T_l):= \sum _{a_i \in \text {S}_P} \sum _{a_j \in \text {S}_L}\delta \left( \tau (a_i),T_k\right) \delta \left( \tau (a_j),T_l\right) \Theta (d_{\rho } - d_{ij})$, $d_{\rho }$ is the interaction cutoff distance, and $\Theta (\cdot )$ a Heaviside step function which sents positive number to 1, and non-positive to 0.

### The output pooling layer

The output pooling layer is based on a graph-level representation. We pool the node representations for the graph embedding, first. Then we apply the embedding for the affinity prediction. That is,

14 $$\begin{aligned} {\hat{y}}:= \text {MLP}\left( \sum _i a_i^{(L)}\right) . \end{aligned}$$

where $a_i^{(L)}$ is the node representation for atom *i* at the last layer of the PAGA model.

### Optimization objective

The optimization objective of PAGA is to minimize the loss between the predicted interaction matrix ${\tilde{Z}}$ and the ground truth interaction matrix *Z*, as well as the loss between the predicted affinity ${\tilde{y}}$ nd the ground truth affinity *y* [2].

The loss function for interaction matrix is given by

15 $$\begin{aligned} {\mathcal {L}}_b:= \sum _{{\mathcal {G}}_I \in {\mathcal {D}}}\Vert \text {F}({\tilde{Z}}) - \text {F}(Z)\Vert , \end{aligned}$$

where $\text {F}(\cdot )$ is the flatten operation for matrix and ${\mathcal {D}}$ is the training set. The loss function for addinity prediction is

16 $$\begin{aligned} {\mathcal {L}}_a:= \sum _{{\mathcal {G}}_I \in {\mathcal {D}}}|{\hat{y}} - y|. \end{aligned}$$

Then the overall optimization formulated as

$$\begin{aligned} {\mathcal {L}}:= {\mathcal {L}}_a + \lambda {\mathcal {L}}_b, \end{aligned}$$

where $\lambda$ is a hyper-parameter that controls the trade-off between the two loss terms.

## Experiment

The publicly available standard PDBbind-v2016 dataset^1^ is used to train and validate our module. This dataset contains a total of 13,283 protein-ligand complexes, with experimental binding affinities expressed as the negative logarithm $pk_a$ of the determined value (e.g, $-\text {log}K_d$, $-\text {log}K_i$, $-\text {log}IC_{50}$). The dataset is hierarchically structured into three nested sets: the General set, the Refined set, and the Core set, with 13,283, 4057, and 290 complexes, respectively. The Core set is used as the test set, a randomly selected subset of 1000 complexes from the difference between the Refined set and the Core set is used as the validation set. The remaining 11,993 complexes in the General set are used as the training set [11, 55].

### Evaluation metrics

To evaluate the performance of our model, we employe four metrics that are widely adopted in computational biology to quantify the accuracy and precision of predictive models: Root Mean Square Error (RMSE), Mean Absolute Error (MAE), Pearson’s correlation coefficient (R), and the standard deviation (SD) in regression [2, 11, 35]. RMSE and MAE provide measures of the average error between predicted and actual values, whereas R and SD are used to assess the correlation and dispersion of the predicted values, respectively. The detail is introduced in Additional file 1. We selecte these metrics to comprehensively evaluate the performance of our model on the test data.

### Baselines

To demonstrate the effectiveness of our CurvAGN model, we compare it against several representative methods from different categories, including free-spatial structure methods, 3D coordinate-based methods, distance-based methods, and angle-distance based methods.

- Free-spatial structure methods: only consider the topological structure of protein-ligand complexes and neglect the spatial structure and interaction information.
  - GraphDTA [56] includes four different variants based on different types of GNNs (GCN, GAT, GIN, and GAT-GCN).
- 3D coordinate-based methods:directly utilize atomic coordinates based on GNNs.
  - SGCN [17]: is based on GCN.
- Distance-based methods: learn graph representation by employing distance information.
  - MAT [33]: learns graph representation by employing a molecule-augmented attention mechanism with the inter-atomic distances.
  - CMPNN [34]: is an edge-oriented model that strengthens the message interactions between edges (bonds) and nodes (atoms) while propagating the distance information.
  - GNN-DTI [13]: leverages GAT to represent a protein-ligand complex graph constructed by the distance between atoms.
  - ELGN [35]: considers distance information and long-distance interaction information between molecules, as well as the topology information of bonds
- Angle-distance based methods: mploy angle and distance information in GNNs.
  - DimeNet [19]: employs the angle and distance information in graph neural network.
  - SIGN [2]: improves GNNs to model the 3D-structure of a protein-ligand complex by not only encoding angle and distance information, but also handling interactions in the complex.

### Implementation details

Let protein set be $S_P:=\{\text {C},\text {N},\text {O},\text {S}\}$ and ligand set be $S_L:=\{\text {C},\text {N},\text {O},\text {S}, \text {P},\text {I},\text {Cl},\text {B},\text {F}\}$, we construct the complex interaction graph and interaction matrix by setting cutoff-threshold $d_{\theta } = 5$ and the interaction cutoff distance $d_{\rho } = 12$ as previous work [8, 57].

For initial node features, we follow the approach in [2, 11], where an atom is represented by an 18-dimensional vector (refer to Table 1 in previous work). To distinguish between ligand and protein atoms, we encode an atom using a 36-dimensional vector, where the first half represents raw features and the second half are all zeros for a ligand and vice versa for a protein atom. The initial edge features consist of vectors of 26 dimensions, where the 26th dimension represents the Euclidean distance between the atoms of the edge, and the first 50 dimensions are filter Forman curvatures with filtration values set as $\{0.1*i:i=0,1,2,\ldots ,49\}$.

**Table 1:** The list of atom features

Atom type	C, N, O, S, P, I, Cl, B, F (onehot)
Atom hybridization	1, 2, 3 (integer)
Number of heavyatoms attached	(integer)
Number of heteroatoms attached	(integer)
SMARTS patterns	Hydrophobic, aromatic, acceptor, donor and ring (onehot)
Partial charge	(float)

The distance and curvature embedding dimensions are both set to 128. Each vector undergoes transformation matrix action, resulting in an embedding of 128 dimensions. To train the model, The Adam optimizer with a learning rate of 0.001 and the batch size of 32 is used to train the model. The dropout rate is set to 0.2, and the hyper-parameter $\gamma$ is set to = 1.75. In the PAGA layers, there are 8 attention heads and 6 angle domains. We list the all settings as following Table 2.

**Table 2:** The parameter setting for our Curv-SIAGN model

Learning rate	Batch size	Hyper-parameter	Cutoff for graph	Dim of Node_emb
0.001	32	$\lambda =1.75$	$\theta _d=5$	128
Heads	Dim of Edge_emb	Cutoff for matrix	Filtrations	Angle domains
4	128	$\theta _{\rho } = 12$	50	6
Dropout	Blocks	Dim of curvature		
0.2	4	128		

All the experiments are conducted on one NVIDIA GeForce RTX 2080 Ti GPU and Inter Xeon Gold 5218 16-Core Processor. And the performance of all the baselines refers to [2].

**Table 3:** The performace comparision on PDBbind-v2016 core set

Methods		RMSE$\downarrow$	MAE$\downarrow$	SD$\downarrow$	R$\uparrow$
Free-spatial	GCN	1.735 (0.034)	1.343 (0.037)	1.719 (0.027)	0.613 (0.016)
	GAT	1.765 (0.026)	1.354 (0.033)	1.740 (0.027)	0.601 (0.016)
Methods	GIN	1.640 (0.044)	1.261 (0.044)	1.621 (0.036)	0.667 (0.018)
	GAT-GCN	1.562 (0.022)	1.191 (0.016)	1.558(0.018)	0.697 (0.008)
Coordinate	SGCN	1.583 (0.033)	1.250 (0.036)	1.582 (0.320)	0.686 (0.015)
	MAT	1.457 (0.037)	1.154 (0.037)	1.445 (0.033)	0.747 (0.013)
Distance	GNN-DTI	1.492 (0.025)	1.192 (0.032)	1.471 (0.051)	0.736 (0.021)
Methods	CMPNN	1.408 (0.028)	1.117 (0.031)	1.399 (0.025)	0.765 (0.009)
	ELGN	1.285 (0.027)	1.013 (0.022)	1.263(0.026)	0.810 (0.012)
Angle	DimeNet	1.453 (0.027)	1.138 (0.026)	1.434 (0.023)	0.752 (0.010)
Methods	SIGN	1.316 (0.031)	1.027 (0.025)	1.312(0.035)	0.797 (0.012)
Ours	CurvAGN	**1.217 (0.012)**	**0.930(0.014)**	**1.191(0.015)**	**0.8305 (0.004)**

Source: We present the average (standard deviation) across 5 random runs, highlighting the best results. Note that the upward arrow $\uparrow$ indicates that a higher value is better, while the downward arrow $\downarrow$ indicates that a higher value is worse

### Performance evaluation

We conduct a comparison of our CurvAGN model and baseline models on the PDBbind v2016 core set. The average and standard deviation of four indicators for testing performance, obtained from five random runs, are presented in Table 3. Overall, the results show that the CurvAGN model outperforms all other models in the dataset.

According to [2, 35], the performance of protein-ligand binding affinity prediction models is heavily influenced by their ability to utilize the spatial structure of protein-ligand complexes. GraphDTA models, which do not use spatial structure, perform poorly. SGCN, which leverages atom coordinates, performs better than the GCN, a variant of GraphDTA. However, SGCN’s performance suffers because its coordinate operations are not rotation invariant. GNN-DTI, with distance information, clearly improves performance over GAT. Among distance-based methods, ELGN and CMPNN focus more on message communication between nodes and edges, resulting in better performance than MAT and GNN-DTI. ELGN leverages long-range intermolecular interactions and incorporates the topology information of bonds, resulting in the best performance among these distance-based methods.

DimeNet is capable of learning the angle and distance structure and outperforms SGCN marginally. SIGN, although it considers angle information, lacks the topology of edges, which could be the main reason for its weaker performance compared to ELGN. Our proposed CurvAGN, on the other hand, captures more spatial information in the form of curvature and utilizes an adaptive graph attention mechanism, resulting in superior performance compared to SIGN.

### Ablation analysis

To validate the importance of multi-scale curvature, heterophily, and multi-head GAT on predicting protein-ligand binding affinity, we compare CurvAGN and its variants on the test data.

**Fig. 2:**
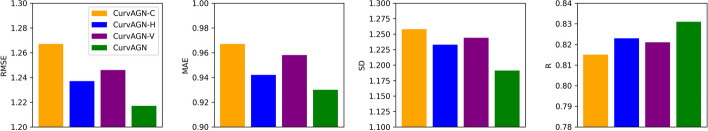
The variants of the CurvAGN model. Different colors mark different models. CurvAGN (green) performs the best, followed by CurvAGN-H (blue) and CurvAGN-V (purple). CurvAGN-C (orange) performs the worst, which suggests that curvature features have a significant impact on protein-ligand binding

- CurvAGN-C: uses the adaptive GAT layer without curvature information.
- CurvAGN-H: uses the vanilla multi-head GAT layer.
- CurvAGN-V: uses the adaptive GAT layer.

As can be observed in the Fig. 2, CurvAGN performs best among all the variants, proving the necessity of curvature information, heterophily and multi-head GAT in predicting protein-ligand binding affinity. Specifically, CurvAGN-C performs worse than CurvAGN because it fails to capture the curvature information. CurvAGN-H suffers from the lack of heterophily, which leads to a performance drop. The different attributes of nodes have varying impacts on the interactions between neighboring nodes. CurvAGN-V fails to capture this, resulting in a decrease in performance. CurvAGN-C has a larger prediction error than CurvAGN-H and CurvAGN-V, indicating that curvature information plays a greater role in improving the model’s performance.

**Fig. 3:**
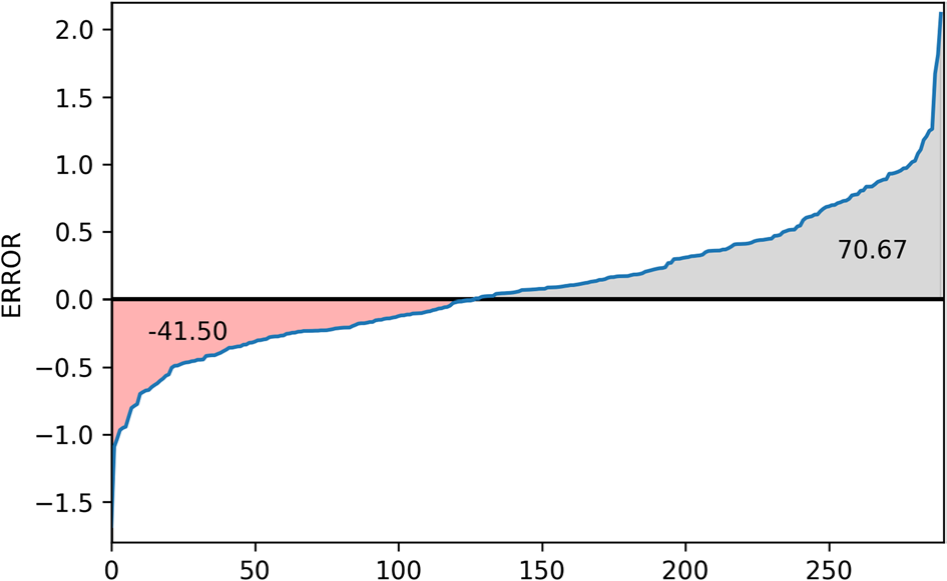
Gains made by CurvAGN on each complex in the test set. The *x*-axis denotes the complexes, and the *y*-axis denotes the error between the difference in absolute prediction error between SIGN and CurvAGN on each complex. The area under curve represents the total gains made by CurvAGN on the test set. The figure shows that our method is only effective for some specific complexes

To check whether the gains made by our method are uniformly distributed across all these 290. We compare the average absolute prediction error of the SIGN and CurvAGN models on the test set across 5 random runs, and the distribution of the difference in absolute prediction error between SIGN and CurvAGN on these complexes is shown in the Fig. 3. In the Fig. 3, the x-axis represents complexes, the y-axis denotes average absolute prediction error, and the area under the curve represents the difference in the total sum of absolute errors between SIGN and CurvAGN on the test set. It is easy to see that the area under the curve above the x-axis (70.67) is greater than the area under the curve below the x-axis (41.50). This implies that CurvAGN performs better than SIGN on average. However, the gains of CurvAGN are not consistent across all complexes, as there are 127 samples with negative y-coordinates.

We compare well-performing complexes with poorly-performing complexes and find our model performs better for complexes with a high ratio of the number of ligand-protein atom pairs with a distance less than 4.8$\text{\AA }{}$ to the total number of ligand-protein atom pairs. This may suggest that intramolecular interactions within the protein and the ligand interfere with the prediction. Further research and analysis is introduced in Additional file 1.

## Conclusion

In this work, we propose CurvAGN, a curvature-based GNN model to predict protein-ligand binding affinity with improved performance, through incorporating the fine-grained geometric information, interaction information among atoms, and heterophily in the complex graph for enhanced representation learning. We first design a curvature block that encodes multiscale curvature information. We then introduce a polar-inspired adaptive graph attention block (PAGA) to capture the heterophily in the complex graph and also the angle and distance information. Additionally, since node attributes rely on the graph structure differently, we use vector attention in the edge2edge layer of PAGA which allows the model to learn different attention weights for different attributes in the node. Additionally, since node attributes rely on the graph structure differently, we use vector attention in the edge2edge layer of PAGA which allows the model to learn different attention weights for different attributes in the node. We train the model on the standard PDBbind-v2016 dataset and its experimental result outperforms SIGN by 7.5% in RMSE and 9.4% in MAE which confirms that the proposed CurvAGN model is effective in improving protein-ligand binding affinity prediction.

For protein-ligand binding affinity prediction, the accuracy of the prediction is important for the design and development of drugs, understanding protein function and interaction mechanisms, etc. Therefore, even if the lift in RMSE is small, our method can improve the accuracy of the prediction and provide more reliable and useful results.

### Future research

We believe that further exploration is warranted to address the issue that our model may not improve prediction accuracy for all protein-ligand complexes. This investigation cannot only reveal the applicability range of our model but also provide new insights for its further improvement. Additionally, we aim to incorporate the overall geometric information of the complexes, such as topological information, into our network structure. Finally, we aspire to apply our model to other areas of biology, such as miRNA-disease association prediction [58] and drug repositioning [59].

### Supplementary Information

**Additional file 1.** Supplemental provides details of valuation metrics used in this work and the relation between complex structure and model performance.

## Data Availability

We use the publicly available standard PDBbind-v2016 dataset http://www.pdbbind.org.cn/.

## Footnotes

1 http://www.pdbbind.org.cn/.

## Declarations

### Code available

Code can be found from this link https://github.com/tumancao/CurvAGN.
