# Supplementary material for: CurvAGN: Curvature-based Adaptive Graph Neural Networks for Predicting Protein-Ligand Binding Affinity
Source: BMC Bioinformatics. 2023 Oct 5;24:378. doi: 10.1186/s12859-023-05503-w (PMC10557336; doi:10.1186/s12859-023-05503-w)
Supplement: Supplementary file 1 — Additional file 1. Supplemental provides details of valuation metrics used in this work and the relation between complex structure and model performance. [file 12859_2023_5503_MOESM1_ESM.pdf]

Supplemental provides details of valuation metrics used in this work and the relation between complex structure and model performance.

Supplemental

Measures We detail the four valuation metrics used in this work. Let y_i be the experimentally measured binding affinity of the i-th complex in dataset $\mathcal{D}$ with n complexes. and $\hat{y}_i$ be the predicted value of y_i.
Root Mean Square Error (RMSE), Mean Absolute Error (MAE) are defined as:

$$\text{RMSE} := \sqrt{\frac{1}{n}\sum_{i=1}^{n}(y_i - \hat{y}_i)^2}, \qquad \text{MAE} := \frac{1}{n}\sum_{i=1}^{n}|y_i - \hat{y}_i|.$$

MAE and RMSE compute the average of the errors between the true affinities and the predicted affinities.
Pearson correlation coefficient (R) is

$$\text{R} := \frac{\sum_{i=1}^{n}(\hat{y}_i - \bar{\hat{y}})(y_i - \bar{y})}{\sqrt{\sum_{i=1}^{n}(\hat{y}_i - \bar{\hat{y}})^2 (y_i - \bar{y})^2}}$$

where $^-$denotes the average value operation. R measures the linear correlation between the true affinities and the predicted affinities.
The standard deviation (SD) is defined as follows:

$$\text{SD} := \sqrt{\frac{1}{n-1}\sum_{i=1}^{n}(y_i - (a + b\hat{y}_i)^2)}$$

where a and b are the intercept and the slope of the regression line, respectively, between the predicted and true affinities. respectively. SD exhibits the average distance of the true affinities and the regression line.
The preference for these evaluation metrics is not consistent when selecting the best model from the two. This can be easily seen from the following example. Let $y = (1, 2, 3)$ be a true values. Assuming $\hat{y}^1 = (1, 1, 1.2)$ and $\hat{y}^2 = (2, 3, 4)$ are the predicted values of two different models. MAE and RMSE of the predicted values are

$$\text{MAE}^1 = \frac{14}{15}, \qquad \text{MAE}^2 = 1,$$

$$\text{RMSE}^1 = \sqrt{\frac{106}{75}}, \qquad \text{RMSE}^2 = 1.$$

The result shows RMSE tends to favor $\hat{y}^2$, while MAE chooses $\hat{y}^1$.

Structure and performance As shown in Figure 3, our model only outperforms SIGN on a subset of protein-ligand complexes. It would be interesting to understand the structural reasons for these differences.

We select 10 complexes from each of the two categories (those on which our model performs better than SIGN and those on which it performs worse) and compare their structural differences, as shown in the table 1. In the table, The first half of the table shows compounds where our model performs poorly, and the second half shows compounds where our model performs well. Let n_1/n denote the ratio of the number of ligand-protein atom pairs (n_1) with a distance less than $4.8\AA$ to the total number of ligand-protein atom pairs (n). from the table, Our model performs better than SIGN when $n_1/n \geq 0.93$, and performs worse when $n_1/n \leq 0.88$.

To investigate whether the prediction of our model is affected by the intermolecular forces within the protein molecule or the drug molecule, we observe the ratio of the number of selected protein-ligand atom pairs (n_1) to the number of atom pairs in the complex (w), and the average number (n_1/m) of protein-ligand atom pairs per atom, where m is the number of atoms in the complex. When $n_1/w < 0.150$, our model performs worse than SIGN, but when $n_1/w \geq 0.150$, our model performs better than SIGN in most cases. When $n_1/m > 2.25$, our model performs better than SIGN, but when $n_1/m \leq 2.25$, our model performs worse in most cases. This suggests that molecular internal information affects the performance of our model.

Table 1: Protein-ligand complex structure and model performance.

Name	Ligand Atoms	Protein Atoms	n_1/n	n_1/w	n_1/m
2wtv	34	91	0.850	0.123	1.864
3bgz	24	57	0.856	0.146	2.050
4w9i	33	78	0.857	0.125	2.090
3jya	16	54	0.858	0.141	1.814
4ih5	17	52	0.862	0.142	1.899
2xdl	16	63	0.863	0.142	1.911
3up2	28	84	0.866	0.144	2.250
3p5o	30	54	0.869	0.153	2.214
4ddk	12	60	0.870	0.133	2.042
1gpn	19	73	0.871	0.141	2.207
3twp	10	39	0.939	0.153	2.204
3nw9	38	121	0.942	0.150	2.648
3arv	25	31	0.946	0.203	3.75
1e66	21	76	0.944	0.174	2.794
3b65	25	96	0.945	0.154	2.256
3arq	36	55	0.944	0.167	2.967
1o5b	13	58	0.948	0.167	2.549
2wbg	22	78	0.942	0.161	2.600
2cet	22	72	0.952	0.151	2.521
4mrz	11	31	0.964	0.217	2.524

Here, n_1, n, w, and m denote the number of ligand-protein atom pairs with a distance less than $4.8\mathring{A}$, the total number of ligand-protein atom pairs, the number of atom pairs in the complex, and is the number of atoms in the complex.
